# Investigating the reaction and substrate preference of indole-3-acetaldehyde dehydrogenase from the plant pathogen *Pseudomonas syringae Pto*DC3000

**DOI:** 10.1042/BSR20202959

**Published:** 2020-12-16

**Authors:** Kaleena Zhang, Josephine S. Lee, Regina Liu, Zita T. Chan, Trenton J. Dawson, Elisa S. De Togni, Chris T. Edwards, Isabel K. Eng, Ashley R. Gao, Luis A. Goicouria, Erin M. Hall, Kelly A. Hu, Katherine Huang, Alexander Kizhner, Kelsie C. Kodama, Andrew Z. Lin, Jennifer Y. Liu, Alan Y. Lu, Owen W. Peng, Erica P. Ryu, Sophia Shi, Maria L. Sorkin, Patricia L. Walker, Grace J. Wang, Mark C. Xu, Rebecca S. Yang, Barrie Cascella, Wilhelm Cruz, Cynthia K. Holland, Sheri A. McClerkin, Barbara N. Kunkel, Soon Goo Lee, Joseph M. Jez

**Affiliations:** 1 Department of Biology, Washington University in St. Louis, St. Louis, MO 63130, U.S.A.; 2 Department of Biology, Williams College, Williamstown, MA 01267, U.S.A.; 3 Department of Molecular Genetics and Cell Biology, University of Chicago, Chicago, IL 60637, U.S.A.; 4 Department of Chemistry and Biochemistry, University of North Carolina Wilmington, Wilmington, NC 28403, U.S.A.

**Keywords:** aldehyde dehydrogenase, auxin, crystal structure, NAD, Pseudomonas syringae, reaction mechanism

## Abstract

Aldehyde dehydrogenases (ALDHs) catalyze the conversion of various aliphatic and aromatic aldehydes into corresponding carboxylic acids. Traditionally considered as housekeeping enzymes, new biochemical roles are being identified for members of ALDH family. Recent work showed that AldA from the plant pathogen *Pseudomonas syringae* strain *Pto*DC3000 (*Pto*DC3000) functions as an indole-3-acetaldehyde dehydrogenase for the synthesis of indole-3-acetic acid (IAA). IAA produced by AldA allows the pathogen to suppress salicylic acid-mediated defenses in the model plant *Arabidopsis thaliana*. Here we present a biochemical and structural analysis of the AldA indole-3-acetaldehyde dehydrogenase from *Pto*DC3000. Site-directed mutants targeting the catalytic residues Cys^302^ and Glu^267^ resulted in a loss of enzymatic activity. The X-ray crystal structure of the catalytically inactive AldA C302A mutant in complex with IAA and NAD^+^ showed the cofactor adopting a conformation that differs from the previously reported structure of AldA. These structures suggest that NAD^+^ undergoes a conformational change during the AldA reaction mechanism similar to that reported for human ALDH. Site-directed mutagenesis of the IAA binding site indicates that changes in the active site surface reduces AldA activity; however, substitution of Phe^169^ with a tryptophan altered the substrate selectivity of the mutant to prefer octanal. The present study highlights the inherent biochemical versatility of members of the ALDH enzyme superfamily in *P. syringae.*

## Introduction

Aldehyde dehydrogenases (ALDHs^1^) are NAD(P)(H)-dependent enzymes found in prokaryotes and eukaryotes that convert a range of aldehyde substrates into their corresponding carboxylic acids [1–4]. Typically, ALDHs are considered housekeeping enzymes with their biochemical function associated with the general detoxification of reactive aldehydes generated through cellular metabolism [5,6]. The inherent biochemical versatility of the ALDHs allow different members of this enzyme superfamily to contribute to a range of metabolic processes beyond detoxification of aldehydes, including ethanol metabolism [7–9], polyamine metabolism [10], plant cell wall ester synthesis [11,12], metabolism of compounds linked to responses to cellular stresses [13,14], and xenobiotic metabolism [15–17]. Recent studies also suggest that in the plant pathogenic microbe *Pseudomonas syringae* strain *Pto*DC3000 (*Pto*DC3000) an ALDH family member synthesizes indole-3-acetic acid (IAA), the primary auxin hormone of plants, to manipulate host plant auxin responses to promote pathogenicity [18].

*P. syringae* produces a variety of virulence factors, including phytohormones or chemical mimics of hormones, to manipulate hormone signaling in its host plants as a means of suppressing disease responses and promoting infection [19–21]. Production of the auxin hormone IAA by *P. syringae* and other plant-associated microbial pathogens is implicated in pathogen virulence [18,22–26]. The plant pathogenic *Pto*DC3000 strain synthesizes IAA through the activity of an indole-3-acetaldehyde dehydrogenase [18]. Earlier work by Xie et al. [27] reported a mutation in *Azospirilum brasilense* that decreased IAA levels in the microbe and was shown to affect a gene encoding an ALDH. Subsequent examination of *Pto*DC3000 identified three ALDHs—AldA (UniProt: PSPTO_0092), AldB (UniProt: PSPTO_2673), and AldC (UniProt: PSPTO_3644)—that are related by 30–40% amino acid identity [18].

AldA from *Pto*DC3000 was determined to function as an indole-3-acetaldehyde dehydrogenase through comprehensive metabolic, biochemical, structural, and in planta analyses [18]. AldA was shown to be essential for synthesis of the phytohormone IAA and as a factor contributing to the virulence of *Pto*DC3000 [18]. Although not as metabolically or kinetically efficient as AldA for auxin synthesis, AldB may also contribute to IAA production in this strain [18].

**Figure 1:**
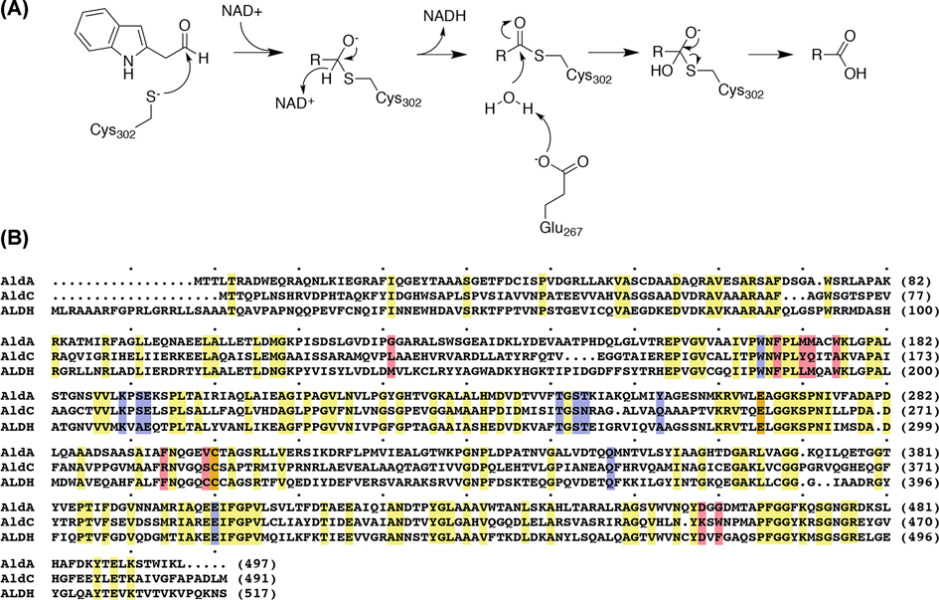
AldA indole-3-acetaldehyde dehydrogenase reaction and sequence comparison (**A**) Proposed mechanism for IAA synthesis catalyzed by AldA. The general reaction for conversion of an aldehyde into a carboxylic acid applies to other substrates of AldA, including octanal. (**B**) Sequence comparison of AldA (AAO53646.1) and AldC (AAO57114.1) from *P. syringae* strain *Pto*DC3000 and human ALDH (P05091.2). Residues corresponding to the catalytic, NAD(H) binding, and substrate binding sites of AldA are highlighted in orange, blue, and red, respectively. Invariant residues are highlighted in yellow.

Biochemical and structural studies of AldC from *Pto*DC3000 indicate that this enzyme functions as a long-chain aliphatic ALDH and does not contribute to pathogenicity [18,28]. Structural studies of AldA and AldC from *Pto*DC3000 indicate that both proteins are members of the larger ALDH enzyme superfamily and share a common chemical reaction mechanism, as well as conserved catalytic residues and NAD(H) binding sites with other such enzymes, including human ALDH, but with differences in the substrate binding site (Figure 1) [18,28].

To better understand the biochemical function of the AldA indole-3-acetaldehyde dehydrogenase from *Pto*DC3000, we examined the contribution of putative active site residues (Cys^302^ and Glu^267^) on the reaction catalyzed by AldA using a series of site-directed mutants. The 2.85 Å resolution X-ray crystal structure of the catalytically inactive AldA C302A mutant in complex with IAA and NAD^+^ reveals that the nicotinamide cofactor adopts a different conformation from that in the previously reported wild-type AldA structure [18]. These structures suggest that AldA undergoes isomerization of the cofactor, as reported for human ALDH [29–30]. Site-directed mutagenesis of the substrate binding site identified Phe^169^ as a determinant of substrate selectivity with the AldA F169W mutant shifting substrate preference toward that of AldC, which prefers the 8-carbon aliphatic aldehyde octanal. These are the first biochemical studies examining the structure–function relationship in the AldA indole-3-acetaldehyde dehydrogenase from *Pto*DC3000 and highlight a conserved reaction chemistry and versatility of the active site for accepting a range of substrates.

## Materials and methods

### Cloning and site-directed mutagenesis

The pET28a-AldA construct was previously described [18]. Site-directed mutants of AldA (catalytic residues: C302A, E267Q, E267A; substrate-binding site: G123L, F169A, F169W, M172A, M173A, W176A, F296A, V301A, D459K, G461W) were generated using the QuikChange PCR method (Agilent Technologies) with the pET28a-AldA construct as template. For expression of His_6_-tagged AldA proteins expression, each pET28a-AldA construct was transformed into *Escherichia coli* BL21 (DE3) (EMD Millipore).

### Protein expression and purification

Transformed *E. coli* BL21 (DE3) cells containing either wild-type or mutant AldA construct were grown at 37°C in Terrific broth with 50 μg.ml^−1^ kanamycin until A_600nm_ ∼0.8. Protein expression was induced by addition of 1 mM isopropyl-1-thio-*β*-d-galactopyranoside to the culture and the cells were then grown at 16°C overnight. Cells were harvested by centrifugation (5000×***g*** for 30 min) and the cell pellet resuspended in 50 mM Tris, pH 8.0, 500 mM NaCl, 25 mM imidazole, 10% (v/v) glycerol, and 1% (v/v) Tween-20. The resuspended cells were lysed by sonication with cell debris removed by centrifugation (12000×***g*** for 45 min). The resulting supernatant was loaded on to a 2–4 ml Ni^2+^-nitriloacetic acid (NTA) column (Qiagen). The column was washed with three to five column volumes of 50 mM Tris, pH 8.0, 500 mM NaCl, 25 mM imidazole, and 10% (v/v) glycerol to remove unbound proteins. The His_6_−AldA fusion protein was eluted from the Ni^2+^-NTA column using 50 mM Tris, pH 8.0, 500 mM NaCl, 25 mM imidazole, 10% (v/v) glycerol, and 250 mM imidazole. Eluted His_6_-tagged AldA protein (either wild-type or mutant) was further purified using a Superdex-200 16/60 size-exclusion column (GE Healthcare) equilibrated in 25 mM Hepes (pH 7.5) and 100 mM NaCl. Fractions corresponding to the purified protein were pooled and concentrated to 8–10 mg.ml^−1^. Protein concentrations were determined using the Bradford method with bovine serum albumin as a standard.

### Enzyme assays and steady-state kinetic analysis

Enzymatic activity of wild-type and mutant AldA proteins was measured by monitoring NADH formation (ε340 nm = 6220 M^−1^.cm^−1^; 100 μl volume) at A_340nm_ using an EPOCH2 microplate spectrophotometer (BioTek), as reported previously [18]. Experiments were performed at 25°C in a standard assay mix of 100 mM Tris/HCl (pH 8.0) and 100 mM KCl. Initial activity assays used standard assay conditions with fixed concentrations of NAD^+^ (2 mM) and either indole-3- acetaldehyde (10 mM) or octanal (10 mM). Steady-state kinetic parameters of wild-type and mutant AldA proteins were determined at 25°C in a standard assay mix with fixed NAD^+^ (2.0 mM) and either varied indole-3-acetaldehyde (0–2.5 mM) or varied octanal (0–10 mM). Protein amounts ranged from 0.1 μg for wild-type AldA to 100 μg for less active point mutants. The resulting initial velocity data were fit to the Michaelis–Menten equation, *v* = (*k*_cat_ [S])/(*K*_m_ + [S]), using SigmaPlot.

### Protein crystallography

Protein crystals of the AldA C302A mutant were grown by the hanging drop vapor diffusion method at 4°C. Crystals of the AldA C302A mutant (9.8 mg.ml^−1^) in complex with IAA and NAD^+^ formed grew in drops of a 1:1 mixture of proteins and crystallization buffer, which was 24% (w/v) PEG-1000, 100 mM Tris/HCl (pH 7.0), 2 mM IAA, and 5 mM NAD^+^. Crystals were stabilized in mother liquor with 30% (v/v) glycerol added as a cryoprotectant before flash-freezing in liquid nitrogen for data collection at 100 K. Diffraction data were collected at beamline 19-ID of the Structural Biology Center, Advanced Photon Source, Argonne National Lab. HKL3000 was used to index, integrate, and scale the collected X-ray data [31]. Molecular replacement was used to solve the X-ray crystal structure of AldA using PHASER [32] with the three-dimensional structure of wild- type AldA PDB: 5IUW) [18]. COOT [33] and PHENIX [34] were used for iterative rounds of model building and refinement, respectively. Data collection and refinement statistics are summarized in Table 1. The final model consisted of eight protein monomers in the asymmetric unit, each with IAA and NAD^+^ bound. No waters were added during refinement due to the resolution of the structure. Atomic coordinates and structure factors for the AldA (C302A)•IAA•NAD^+^ complex (PDB: 7JSO) were deposited in the RCSB Protein Data Bank (www.rcsb.org).

**Table 1:** Summary of crystallographic data collection and refinement statistics

Crystal	AldA (C302A)•IAA•NAD^+^
Space group	C2
Cell dimensions	*a* = 333.8 Å, *b* = 161.1 Å, *c* = 85.0 Å
Data collection	
Wavelength	0.979 Å
Resolution range (highest shell)	47.9–2.85 Å (2.92–2.85 Å)
Reflections (total/unique)	555535/103193
Completeness (highest shell)	98.3% (99.8%)
<I/σ> (highest shell)	13.9 (5.4)
R_sym_ (highest shell)	9.2% (24.1%)
Refinement	
R_cryst_/R_free_	21.6%/25.4%
Number of protein atoms	29536
Number of ligand atoms	456
R.m.s. deviation, bond lengths	0.010 Å
R.m.s. deviation, bond angles	1.18°
Avg. B-factor: protein, ligand	35.9, 69.4 Å^2^
Stereochemistry: favored, allowed, outliers	95.8, 4.2, 0%

## Results and discussion

### Mutagenesis of the catalytic cysteine and glutamate residues in AldA

Initial biochemical analysis of AldA showed that this ALDH converts indole-3-acetaldehyde into IAA and shares a conserved three-dimensional structure and active site features with other members of the ALDH enzyme superfamily [18]. As with other ALDHs [4,8,35–37], AldA retains the invariant active site cysteine (Cys^302^) required for formation of a thioacyl-enzyme intermediate in the reaction sequence and a conserved glutamate (Glu^267^) that serves to activate a water molecule for hydrolysis of the intermediate (Figure 1). To probe the role of the corresponding catalytic residues in AldA, the C302A, E267Q, and E267A site-directed mutants were generated. Each mutant was expressed in *E. coli* as a His_6_-tagged protein with yields and a tetrameric oligomerization similar to that of wild-type AldA [18] (Supplementary Figure S1). As reported previously [18], wild-type AldA was active with indole-3-acetaldehyde as a substrate (Table 2). Enzyme assays using the AldA C302A, E267Q, and E267A mutants showed no detectable activity above background rates in assays using up to 1000-fold higher amounts of protein compared with wild-type AldA. This result is consistent with the canonical ALDH reaction mechanism in which a cysteine (Cys^302^ in AldA) reacts with the aldehyde substrate to form an thioacyl-enzyme intermediate, which reacts with NAD^+^ for hydride transfer, followed by hydrolysis of the intermediate via a water molecule activated by a glutamate (Glu^267^ in AldA) and subsequent release of the carboxylic acid product [8,19,35–37].

**Table 2:** Steady-state kinetic parameters of wild-type and mutant AldA proteins

Protein	Indole-3-acetaldehyde			Octanal		
	*k*_cat_ (min^−1^)	*K*_m_ (*μ*M)	*k*_cat_/*K*_m_ (M^−1^.s^−1^)	*k*_cat_ (min−^1^)	*K*_m_ (*μ*M)	*k*_cat_/*K*_m_ (M^−1^.s^−1^)
WT	240 ± 9	0.18 ± 0.02	22,040	96 ± 6	2.7 ± 0.6	593
G123L	202 ± 7	0.26 ± 0.04	12,950	71 ±4	2.5 ± 0.5	473
F169A	88 ± 12	4.7 ± 0.6	312	50 ± 4	>10^*^	83
F169W	41 ± 4	1.6 ± 0.4	427	108 +13	0.69 ±0.10	2609
M172A	220 ± 23	0.92 ± 0.11	3986	92 ± 4	2.7 ± 2.5	567
M173A	184 ± 23	1.5 ± 0.4	2044	63 ± 9	>10^*^	105
W176A	10 ± 2	8.8 ± 2.5	19	-	-	-
F296A	18 ± 1	7.0 ± 2.1	43	8	>10^*^	13
V301A	81 ± 10	7.2 ± 0.9	190	32 ± 9	>10^*^	54
D459K	72 ± 14	0.86 ± 0.23	1395	–	–	–
G461W	160 ± 14	0.29 ± 0.05	9195	98 ± 5	4.1 ± 0.8	398

The protein and location of the mutation are indicated.

Enzyme assays were performed as described in the ‘Materials and methods’ section.

Average values ± S.D. (*n*=3) are shown.

* Estimated *K*_m_ greater than 10 mM solubility of substrate tested.

### Structure of AldA C302A mutant in complex with IAA and NAD^+^ and cofactor isomerization

**Figure 2:**
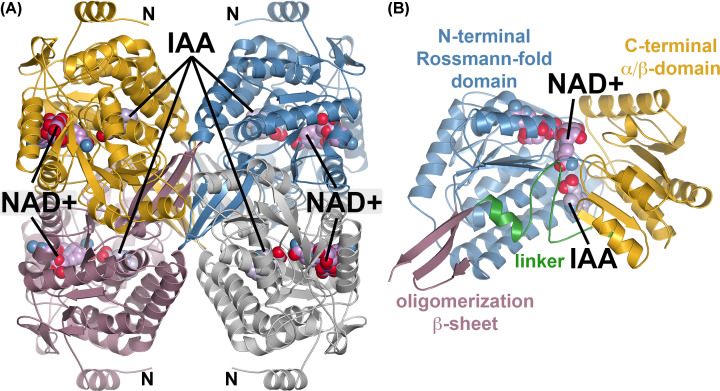
Overall three-dimensional structure of the AldA(C302A)•NAD^+^•IAA complex (**A**) Tetrameric assembly of the AldA C302 mutant. Each monomer is colored individually with the locations of bound NAD^+^ and IAA indicated. The N-terminus of each monomer is also noted. (**B**) Domain organization of an AldA monomer. The view is slightly rotated from that in (A). The N-terminal Rossmann-fold (blue), C-terminal ^α^/*β* domain (gold), oligomerization *β*-sheet (rose), and domain linker (green) are indicated and labeled with ligand positions indicated.

To further examine the reaction mechanism of AldA, the AldA C302A mutant was crystallized in the presence of NAD^+^ and IAA. The 2.85 Å resolution X-ray crystal structure of the AldA C302A mutant in complex with these ligands was solved by molecular replacement (Table 1). The overall three-dimensional structure of the AldA C302A mutant is a tetramer (Figure 2A) with each monomer consisting of an N-terminal NAD(H) binding domain defined by a canonical Rossmann-fold, an interdomain linker, a *β*-strand oligomerization region, and a C-terminal mixed ^α^/*β*-domain that includes the catalytic cysteine and the aldehyde substrate binding site (Figure 2B). The AldA C302A mutant structure is similar to that of the previously reported wild-type protein [18] with a 0.24 Å^2^ r.m.s.d. for 497 C_α_-atoms in the monomer. NAD^+^ was observed bound in the N-terminal NAD(H) binding site and IAA in the C-terminal substrate binding site.

**Figure 3:**
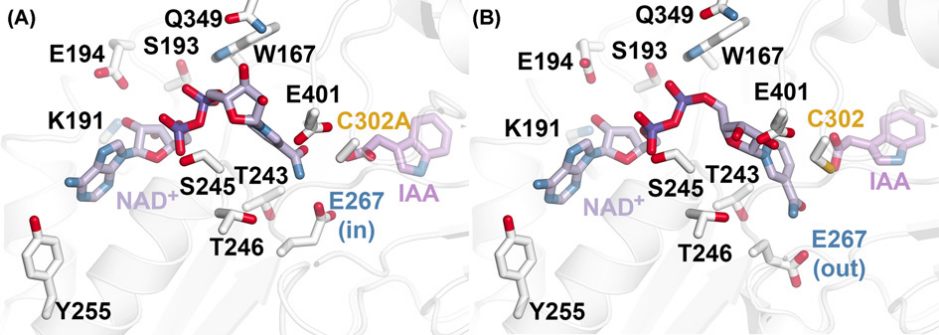
NAD^+^ isomerization in AldA (**A**) NAD^+^ binding in the AldA C302A X-ray crystal structure. The nicotinamide half of the ligand adopts a conformation that positions the nicotinamde ring away from the catalytic site (i.e., C302A in gold), which is ideal for the hydrolysis step of the reaction. The side-chain of Glu^267^ (blue; in) is positioned into the active site. (**B**) NAD^+^ binding in the AldA wild-type X-ray crystal structure. As previously reported [18], the nicotinamide half of the ligand adopts a conformation that positions the nicotinamde ring into the catalytic site (i.e., C302 in gold), which is ideal for the hydride transfer step of the reaction. The side-chain of Glu^267^ (blue; out) is positioned away from the active site.

Although the overall three-dimensional structures of the wild-type and C302A mutant AldA proteins are similar, there is a difference in the observed conformations of NAD^+^ in each structure (Figure 3). As reported previously, residues in the NAD(H) binding site of AldA and other ALDHs are highly conserved (Figure 1B). In the AldA C302A mutant structure, the adenine half of the NAD^+^ molecule is bound in the same orientation as previously reported for the wild-type enzyme [18]. Likewise, the same interactions between the adenine-ribose-pyrophosphate moieties of NAD^+^ and Tyr^255^, Lys^191^, Glu^194^, Ser^193^, Ser^245^, and Trp^167^ are observed in the mutant AldA structure (Figure 3). The nicotinamide half of the coenzyme is bound with the ribose oriented toward Gln^349^ and the nicotinamide ring pulled away from the catalytic site (defined by the C302A mutation) by contraction of the coenzyme structure (Figure 3A). This orientation of NAD^+^ retains a hydrogen bond between the ligand amide group and the carboxylate side-chain of Glu^267^, which is positioned into the AldA active site. In comparison, in the earlier X-ray crystal structure of AldA [18] NAD^+^ adopted an extended conformation with the nicotinamide half of the cofactor placed deeper into the active site and in proximity to Cys^302^ (Figure 3B). This allows Glu^401^ to form a bi-dendate interaction with the hydroxyl groups of the nicotinamide-ribose group. The nicotinamide ring is positioned in proximity to the catalytic cysteine with the amide moiety of NAD^+^ still hydrogen bonding with the carboxylate side-chain of Glu^267^, which shifts outward from the catalytic site (Figure 3B).

Site-directed mutagenesis of the conserved catalytic residues of AldA and the three-dimensional structures of this enzyme imply a shared reaction mechanism with other members of the ALDH superfamily (Figure 1A), including coenzyme isomerization that allows for hydride transfer (Figure 3B) and hydrolysis of the thioacyl-enzyme intermediate (Figure 3A) during the reaction mechanism [29,35]. As for other ALDHs, we suggest that AldA binds indole-3-acetaldehyde and NAD^+^ in its active site and undergoes formation of a thioacyl-enzyme intermediate at Cys^302^ and subsequent hydride transfer (Figure 1A) with the cofactor adopting a compact conformation similar to that observed in the AldA C302A mutant structure (Figure 3). Isomerization of NAD^+^ and movement of Glu^267^ then allows for hydrolysis by an activated water molecule and release of the final IAA product.

### Site-directed mutagenesis of the AldA substrate-binding site and identification of a determinant of substrate selectivity

**Figure 4:**
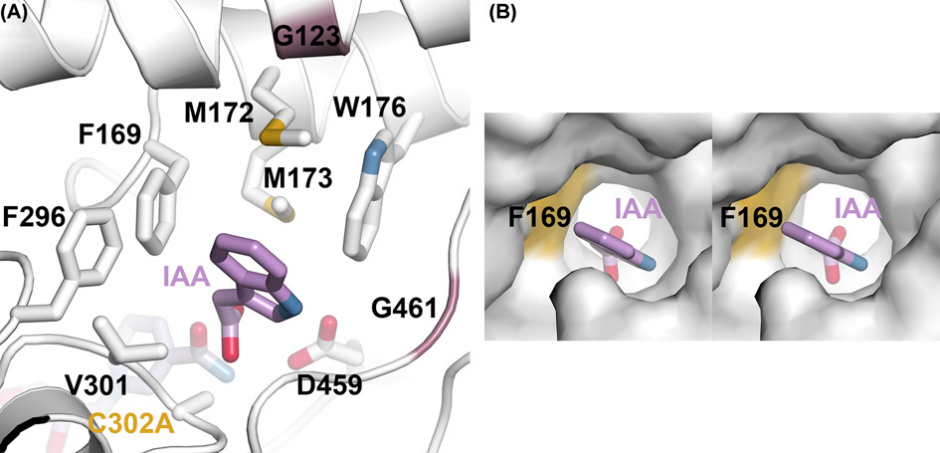
AldA C302A mutant substrate binding site (**A**) IAA binding in the AldA C302A mutant X-ray crystal structure. Residues in the substrate binding site are shown. Two glycine residues in the site are represented by the rose colored secondary structure. (**B**) Surface stereo-view of the AldA substrate binding site. The surface corresponding to Phe^169^ is highlighted in gold.

The three-dimensional structures of both wild-type AldA [18] and the C302A mutant (Figure 4A) provide similar information on how IAA binds in the active site. The IAA carboxylate group is oriented into the catalytic site (i.e., ∼3Å from side-chain of C302A in AldA) with the indole ring positioned by van der Waals interactions in a largely apolar binding site, which includes Gly^123^, Phe^169^, Met^172^, Met^173^, Trp^176^, Phe^296^, Val^301^, Thr^303^, Asp^459^, and Gly^461^ (Figure 4A). To probe the contributions of various residues in the AldA binding site, a series of site-directed point mutants (G123L, F169A, F169W, M172A, M173A, W176A, F296A, V301A, D459K, and G461W) were generated. The G123L, F169W, D459K, and G461W mutants substitute the AldA residue with the corresponding amino acid found in AldC (Figure 1B). Each AldA mutant was expressed as a His_6_-tagged fusion protein in *E. coli* and isolated by affinity chromatography with yields comparable with wild-type.

Using indole-3-acetaldehyde as a substrate (Table 2), AldA variants with mutations in residues closer to the solvent-exposed entrance of the IAA binding site—G123L and G461W—displayed approximately two-fold decreases in catalytic efficiency (i.e., *k*_cat_/*K*_m_). Mutations of Met^172^ (M172A), Met^173^ (M173A), and Asp^459^ (D459K) resulted in 5- to 15-fold reductions in *k*_cat_/*K*_m_ compared with wild-type AldA. The most detrimental substitutions were those of residues in the ‘aromatic box’—Phe^169^, Trp^176^, Phe^296^, and Val^301^. The F169A, F169W, W176A, F296A, and V301A mutants generally exhibited decreased turnover rates and increased *K*_m_ values with roughly 50- to 1150-fold decreases in catalytic efficiency. These results suggest that alterations in the apolar surface of the AldA substrate site likely alter binding of indole-3-acetaldehyde for catalysis.

Recent work identified the AldC enzyme from *P. syringae* strain *Pto*DC3000 as a long-chain aliphatic ALDH [28]. To compare the activity of AldA with AldC, wild-type AldA was assayed using octanal as a substrate; this is the preferred substrate of AldC [28]. Although AldA displayed activity with octanal (Table 2 and Supplementary Figure S2A,B), it was 37-fold less efficient as a substrate compared with indole-3-acetaldehyde. We also used octanal as a substrate for the AldA point mutants described above (Table 2). Two mutants—W176A and D459K—had no detectable activity with this substrate. The F169A, M173A, F296A, and V301A mutants exhibited activity with octanal, but no substrate saturation. This indicates that the potential *K*_m_ values of these mutants are greater than the 10 mM solubility limit of octanal. The AldA G123L, M172A, and G461W substitutions had only modest effect on the catalytic efficiency with octanal compared with wild-type protein. Interestingly, the F169W mutant accepted octanal (*k*_cat_/*K*_m_ = 2610 M^−1^.s^−1^) as the preferred substrate compared with indole-3-acetaldehyde (*k*_cat_/*K*_m_ = 427 M^−1^.s^−1^) (Table 2 and Supplementary Figure S2C,D). This mutation altered substrate selectivity of AldA toward that of AldC by six-fold.

The three-dimensional structure of AldA suggests a possible basis for the change in substrate selectivity of the AldA F169W mutant (Figure 4B). In AldA, Phe^169^ is positioned deep in the ‘aromatic box’ region of the substrate binding site. As described for other ALDHs, residues in this region of the active site form an apolar tunnel that influences substrate selectivity [38–40].

Substitution of Phe^169^ with a larger tryptophan residue likely constricts the entrance to the catalytic center of AldA. The tryptophan side-chain of the F169W mutant may sterically interfere with the indole ring of IAA; whereas, the aliphatic chain of octanal better fits the substrate binding site of the mutant. This would account for the change in selectivity for octanal as a substrate versus indole-3-acetaldehyde in the AldA F169W mutant. Interestingly, the corresponding residue at this position in AldC from *P. syringae*, which prefers octanal versus other aliphatic aldehydes as a substrate, is a tryptophan [28]. Comparison of the *k*_cat_/*K*_m_ for the AldA F169W mutant (2610 M^−1^s^−1^) with that of AldC (924 M^−1^s^−1^; [28]) suggest that the identity of the residue at this position of the substrate binding site of these enzymes is critical for substrate preference.

## Summary

As a widespread enzyme family, ALDHs catalyze conversion of aldehydes into carboxylates using a shared NAD(H)-dependent mechanism [1–4]. The versatility of these enzymes allows them to perform a range of biological roles. For example, in the plant pathogen *P. syringae* uses AldA as an indole-3-acetaldehyde dehydrogenase that contributes to pathogen virulence [18]. Here we further examined the biochemical function of AldA. Site-directed mutagenesis of AldA confirms that the enzyme shares the same catalytic residues (Cys^302^ and Glu^267^) as other ALDHs. As described for other ALDHs, the cysteine reacts with the aldehyde to form a thioacyl-enzyme intermediate, which reacts with NAD^+^ for hydride transfer, followed by hydrolysis of the intermediate via a water molecule activated by a glutamate, and release of the carboxylate (Figure 1) [8,19,35–37].

Structural analysis of the AldA C302A mutant and comparison with the wild-type AldA (Figure 3) reveals that the NAD^+^ undergoes a conformational isomerization, as reported for other ALDHs [29,35], that allows for each half reaction to occur. The other common feature of the ALDH family is the inherent versatility of their substrate binding sites to accommodate a variety of molecules, as these enzymes typically display broad substrate profiles [2–7,38–40]. For example, the ‘aromatic box’ formed by apolar residues in the substrate binding site helps define which aldehydes are recognized by the ALDH [38–40]. Although mutations in the AldA substrate binding site reduced activity with indole-3-acetaldehyde, as well as octanal (Table 2), substitution of Phe^169^ with a tryptophan (the residue at this position found in AldC) enhanced activity with the aliphatic aldehyde. This suggests that key changes in the site are important for the evolution of the diverse biochemical functions in members of the ALDH enzyme superfamily.

## Supplementary Material

Supplementary Figures S1-S2

## Data Availability

The data and material presented in this manuscript are available from the corresponding author on reasonable request. Atomic coordinates and structure factors for the AldA (C302A)•IAA•NAD^+^ complex (PDB: 7JSO) were deposited in the RCSB Protein Data Bank (www.rcsb.org).

## Abbreviations

ALDH: aldehyde dehydrogenase
IAA: indole-3-acetic acid
NTA: nitriloacetic acid
*Pto*DC3000: *Pseudomonas syringae* strain *Pto*DC3000
