# Supplementary material for: Investigating the reaction and substrate preference of indole-3-acetaldehyde dehydrogenase from the plant pathogen Pseudomonas syringae PtoDC3000
Source: Biosci Rep. 2020 Dec 16;40(12):BSR20202959. doi: 10.1042/BSR20202959 (PMC7745063; doi:10.1042/BSR20202959)

Supplemental Information

Supplemental Figure 1. Representative purification of AldA mutant proteins. (A) Size-exclusion chromatographic analysis of the AldA E267A mutant using a Superdex-200 26/60 FPLC column. Elution volume is indicated. (B) Molecular weight calibration of the size-exclusion column. The following standards were used to calibrate the column: ferritin (440 kDa), catalase (232 kDa), aldolase (158 kDa), conalbumin (75 kDa), ovalbumin (44 kDa), carbonic anhydrase (29 kDa), ribonuclease (13.7 kDa), and aprotinin (6.5 kDa). The elution of the AldA E267A mutant from panel A is indicated. (C) Representative SDS-PAGE analysis of mutant AldA. Each lane corresponds to molecular weight standards, *E. coli* lysate, flow-through from the nickel-affinity chromatography loading (FT), after washing the column (wash), and the eluted samples for the E267A and E267Q mutants.

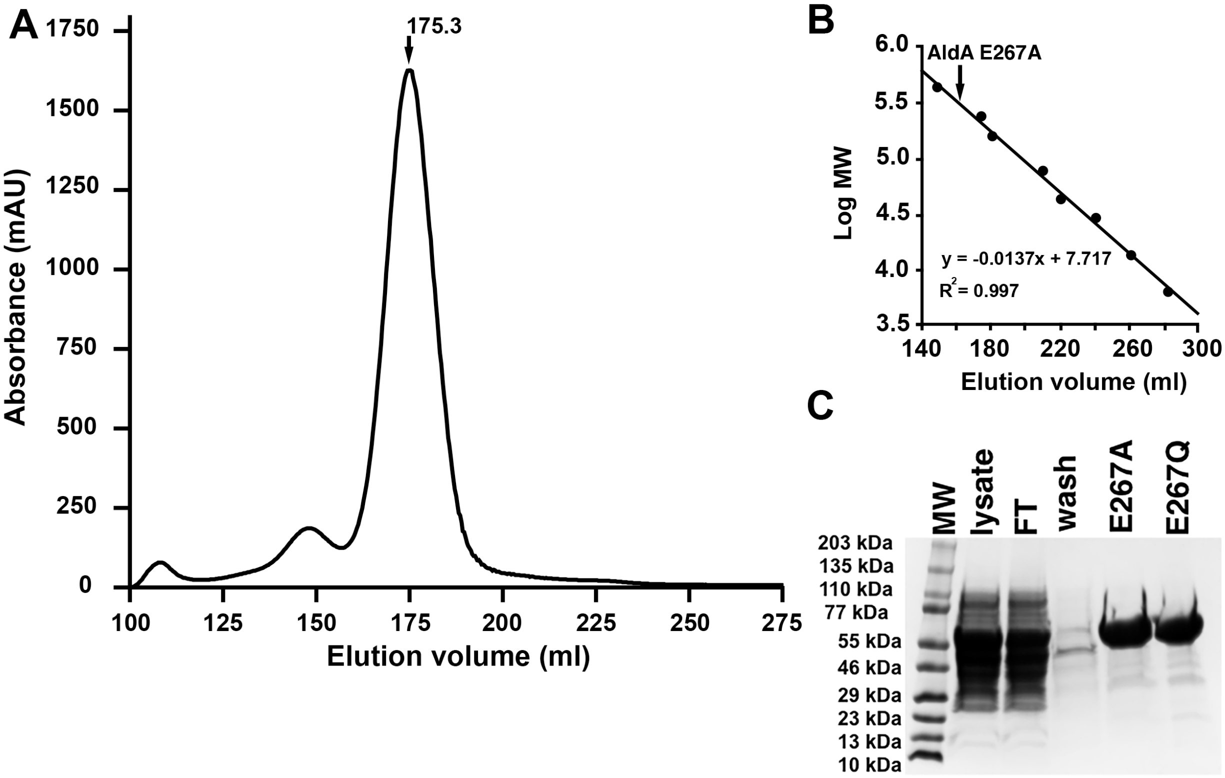

Supplemental Figure 2. Representative initial velocity versus substrate concentration data for wild-type AldA using either indole-3-acetaldehyde (A) or octanal (B) as a substrate and the AldA F169W mutant using either indole-3-acetaldehyde (C) or octanal (D) as a substrate. Data were fit to the Michaelis-Menton equation, as summarized in Table 2.

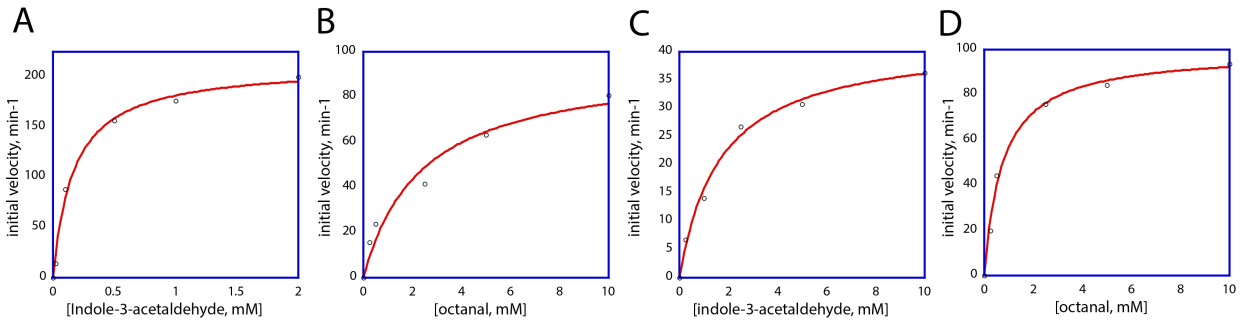

Supplement: Supplementary Figures S1-S2 [file BSR-2020-2959_supp.pdf]
